# Corneal Segmentation Analysis Increases Glaucoma Diagnostic Ability of Optic Nerve Head Examination, Heidelberg Retina Tomograph's Moorfield's Regression Analysis, and Glaucoma Probability Score

**DOI:** 10.1155/2015/215951

**Published:** 2015-05-27

**Authors:** F. Saenz-Frances, L. Jañez, C. Berrozpe-Villabona, L. Borrego-Sanz, L. Morales-Fernández, A. Acebal-Montero, C. D. Mendez-Hernandez, J. M. Martinez-de-la-Casa, E. Santos-Bueso, J. Garcia-Sanchez, J. Garcia-Feijoo

**Affiliations:** Hospital Clínico Universitario San Carlos, 28040 Madrid, Spain

## Abstract

*Purpose*. To study whether a corneal thickness segmentation model, consisting in a central circular zone of 1 mm radius centered at the corneal apex (zone I) and five concentric rings of 1 mm width (moving outwards: zones II to VI), could boost the diagnostic accuracy of Heidelberg Retina Tomograph's (HRT's) MRA and GPS. *Material and Methods*. Cross-sectional study. 121 healthy volunteers and 125 patients with primary open-angle glaucoma. Six binary multivariate logistic regression models were constructed (MOD-A1, MOD-A2, MOD-B1, MOD-B2, MOD-C1, and MOD-C2). The dependent variable was the presence of glaucoma. In MOD-A1, the predictor was the result (presence of glaucoma) of the analysis of the stereophotography of the optic nerve head (ONH). In MOD-B1 and MOD-C1, the predictor was the result of the MRA and GPS, respectively. In MOD-B2 and MOD-C2, the predictors were the same along with corneal variables: central, overall, and zones I to VI thicknesses. This scheme was reproduced for model MOD-A2 (stereophotography along with corneal variables). Models were compared using the area under the receiver operator characteristic curve (AUC). *Results*. MOD-A1-AUC: 0.771; MOD-A2-AUC: 0.88; MOD-B1-AUC: 0.736; MOD-B2-AUC: 0.845; MOD-C1-AUC: 0.712; MOD-C2-AUC: 0.838. *Conclusion*. Corneal thickness variables enhance ONH assessment and HRT's MRA and GPS diagnostic capacity.

## 1. Introduction

Whereas stereoscopic assessment of the optic nerve head (ONH) remains the gold standard of structural glaucoma diagnosis, many new automatic devices have been developed to either improve the diagnostic accuracy or help ophthalmic practitioners in routine clinical practice [1–19]. Amongst them, the Heidelberg Retina Tomograph (HRT) has proven to be a very useful tool despite its limitations [20–25].

Although broad evidence suggests that central corneal thickness (CCT) is both an IOP measurement confounder and an independent risk factor for developing primary open-angle glaucoma (POAG), most of the literature regarding this matter only takes into account the very centre of the cornea [25–48]. In order to study noncentral corneal thickness differences between healthy subjects and patients suffering from POAG, we developed a ring-shaped corneal segmentation model [49] which suggested that these differences indeed exist. Moreover, in spite of not being a diagnostic tool in itself, our pattern showed some diagnostic capacity between cases of POAG and healthy controls [49].

The purpose of this study is to establish if the addition of the corneal thickness variables generated through our segmentation model could improve the diagnostic accuracy of the HRT results.

## 2. Material and Methods

We performed a cross-sectional study in 121 healthy volunteers and 125 patients with primary open-angle glaucoma. The patients were recruited from the Glaucoma Unit of the Hospital Clínico San Carlos, Madrid (Spain), and control subjects among the patients' companions and hospital staff. The study protocol was approved by our institution's review board and complied with the guidelines of the Declaration of Helsinki. Informed consent was obtained from each participant before inclusion in the study. All the study participants were Caucasian. Eyes were considered to be glaucomatous if they had shown abnormal results in at least three consecutive visual field exams (Octopus TOP-G1X) and if there was evidence of glaucomatous damage as determined by the appearance of the ONH and retinal nerve fiber layer thickness (RNFL) as determined using Heidelberg Spectralis Optic Coherence Tomograph (OCT) and stereophotographs of the ONH. Only patients with early and moderate glaucoma (according to Hodapp, Parrish, and Anderson's Classification modified for Octopus perimetry) were eligible for the study. The glaucoma patients were required to show gonioscopic evidence of a normal and open angle. Subjects with nonprimary open-angle glaucoma (e.g., pseudoexfoliation, pigment dispersion, and neovascularization) were excluded. It was accordingly checked that control subjects had an IOP < 21 mmHg, a normal visual field, and a nonglaucomatous appearing optic disc and RNFL thickness. With regard to Moorfield's regression analysis (MRA) and glaucoma probability score (GPS), subjects showing a borderline diagnosis of glaucoma were excluded.

General exclusion criteria were a spherical equivalent greater than 5 diopters, or 3 or more diopters of astigmatism, a best corrected visual acuity lower than 20/25, opacities in the cornea or lens impairing optic nerve head visualization, and alterations in optic nerve head morphology, such as oblique discs or peripapillary atrophy. We also excluded subjects who had undergone prior eye surgery and those whose visual field defects were of causes other than glaucoma (e.g., demyelinating disease, nonglaucomatous neuropathy, or a central nervous system disorder). If both eyes of a patient or subject fulfilled all the inclusion and exclusion criteria, the eye to be examined was determined through an automatic randomization procedure (http://www.randomization.com).

**Figure 1:**
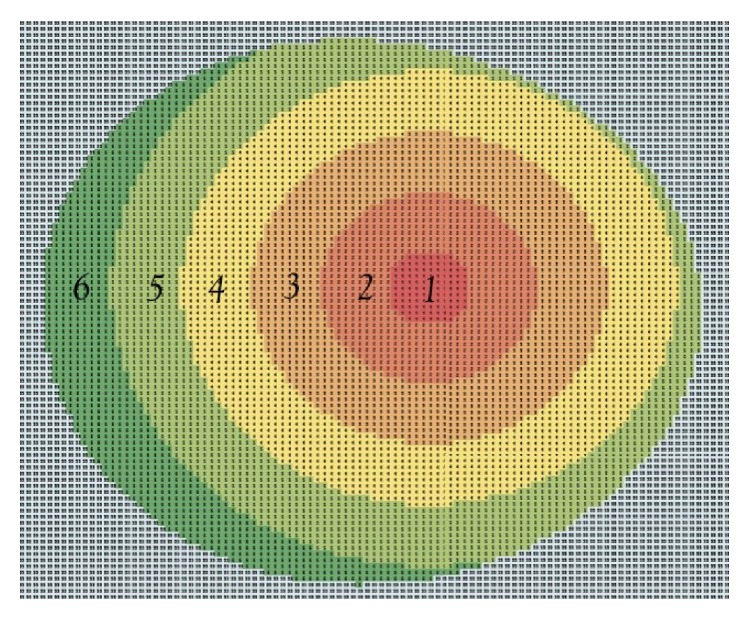
Corneal thickness segmentation scheme into virtual circular zones concentric with the corneal apex (1 to 6 denote zone I to zone VI, resp.).

All the study participants underwent a Pentacam (Pentacam, Oculus USA) examination, ultrasound pachymetry (Dicon P55, Paradigm Medical Industries Inc., Salt Lake City, UT, USA), a Confocal scanning laser tomography (HRT-3, Heidelberg Engineering, Germany), and a Heidelberg Spectralis RNFL thickness OCT (Heidelberg Spectralis, Heidelberg Engineering, Germany). Being noncontact procedures, the Pentacam, HRT, and OCT evaluations were performed first. The order in which the remaining exploration was performed was established through an automatic randomization procedure (http://www.randomization.com). Using the pachymetric maps generated by the Pentacam (this instrument makes thickness measurements across the entire cornea perpendicular to its surface separated by a distance of 1 *μ*m), we virtually segmented the cornea into a central circular zone of radius of 1 mm (designated zone I) centered at the corneal apex (this is the point of maximum curvature or height, typically temporal to the centre of the pupil) and several concentric rings of 1 mm width each with the same centre (5 rings until the corneal limbus denoted zones II, III, IV, V, and VI, resp., moving outward from the centre) (Figure 1).

Six binary multivariate logistic regression models were constructed (denoted as MOD-A1, MOD-A2, MOD-B1, MOD-B2, MOD-C1, and MOD-C2, resp.). In all of them, the dependent variable was the presence (or not) of glaucoma. In the MOD-A1 model the predictor was the result of the analysis of the stereophotography of the optic nerve head (ONH) by an examiner expert in ophthalmology (dichotomous result: glaucoma or not) (the examiner was masked to those who establish the diagnosis of glaucoma or not of the members of the sample according the comprehensive examination described before). In the MOD-B1 and MOD-C1 models the predictor was the result of the MRA and GPS, respectively (dichotomous result: glaucoma or not, as the borderline cases were an exclusion criterion), adjusted for age and disc size (as determined by the HRT); the presence of interactions between age and disc size with the result of MRA and GPS was also studied. In the MOD-B2 and MOD-C2 models the predictors were the result of MRA and GPS, respectively (dichotomous result: glaucoma or not, as the borderline cases were an exclusion criterion), but, in these models, along with corneal variables, central corneal thickness (CCT) (as determined by ultrasound pachymetry), overall corneal thickness (OT), and the thicknesses of zones created with the corneal segmentation (zone I to zone VI) (all adjusted for age and disc size), interactions between age and disc size with all the predictors were studied. The same scheme was reproduced for model MOD-A2: the predictors were the result of the analysis of the stereophotography of the optic nerve head (ONH) by an expert in glaucoma (dichotomous result: glaucoma or not) along with the corneal variables mentioned before (for MOD-B2 and MOD-C2).

We determined the discriminating capacity between glaucoma and normality of each predictor in each model determining its odds ratio (OR) of suffering from glaucoma.

Models were compared using the area under the receiver operator characteristic (ROC) curve (AUC) and the Nagelkerke-*R* ^2^.

Sensitivity, specificity, positive predictive value, and negative predictive value of detecting glaucoma (cutoff of 0.5) were also determined for each model.

Statistical difference between the AUC of each pair of models (MOD-A1 and MOD-A2, MOB-B1 and MOD-B2, and MOD-C1 and MOD-C2, resp.) was established performing several DeLong's tests for two correlated ROC curves (one test for each pair).

The influence on the diagnostic accuracy of each model (as a dichotomous variable: right classification or wrong classification) of the stage of glaucoma was also assessed through several logistic univariate regression models being the predictor the stage of glaucoma (three categories according to Hodapp, Parrish, and Anderson's classification: normality, early stage glaucoma, and moderate glaucoma, as severe glaucoma was an exclusion criterion).

## 3. Results

The two groups examined in this cross-sectional study were 121 eyes of 121 healthy subjects and 125 eyes of 125 patients with POAG.

Table 1 provides the means and standard deviations (sd) of the corneal variables recorded.

Mean age was 64.25 years (sd: 13.71); mean disc size was 2.09 (sd: 0.495).

The normal distribution of data was confirmed by the Kolmogorov-Smirnov and Shapiro-Wilk tests.

**Figure 2:**
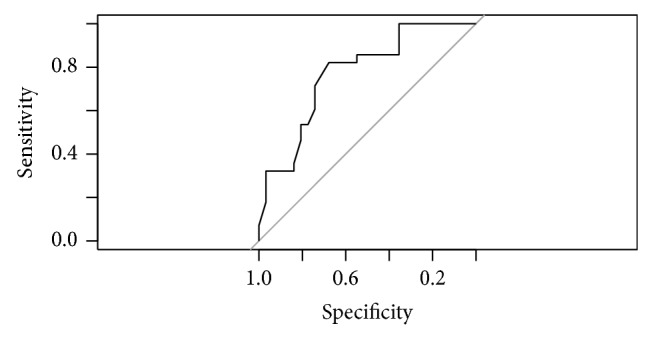
ROC curve for model MOD-A1.

MOD-A1 model revealed that the exam of the ONH by an expert examiner exhibits significant diagnosis ability for glaucoma (OR = 4.83; 95% CI: 1.23–15.44). No significant interaction between age or disc size and the result of the examiner arose. AUC of this model was 0.771 (95% CI: 0.651–0.891). Sensitivity is 67.86%; specificity is 74.19%. Nagelkerke *R* ^2^ is 29,01%.  Figure 2 presents the ROC curve for this model.

**Figure 3:**
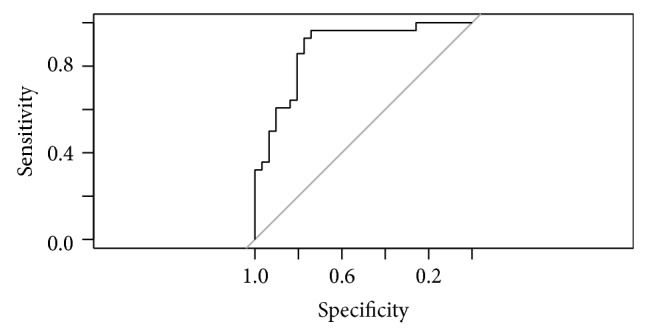
ROC curve for model MOD-A2.

Including corneal variables as predictors (MOD-A2) along with MRA outcome results in a furtherance of the diagnostic capacity of MOD-A1: OR = 9.43; 95% CI: 5.11–12.81. Apart from MRA, other predictors showed discriminating capacity between POAG and normality (Table 2). AUC of this model was 0.88 (95% CI: 0.791–0.969). Sensitivity is 78.57%; specificity is 80.65%. Nagelkerke *R* ^2^ is 52.14%.  Figure 3 presents the ROC curve for this model.

**Figure 4:**
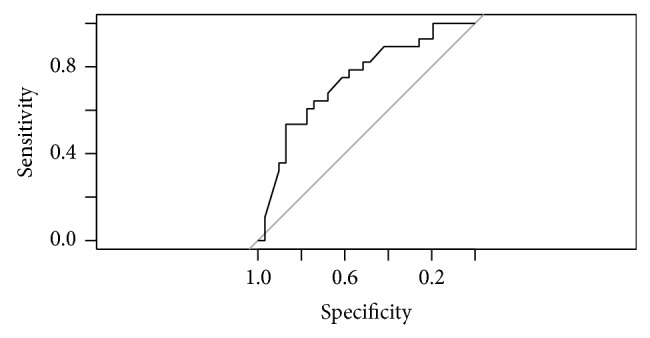
ROC curve for model MOD-B1.

MOD-B1 revealed that also MRA outcome has significant diagnosis capability for glaucoma: OR = 2.15; 95% CI: 1.08–6.04. No significant interaction between age or disc size and the result of Moorfield's regression analysis arose. AUC of this model was 0.736 (95% CI: 0.61–0.739). Sensitivity is 64.29%; specificity is 73.28%. Nagelkerke *R* ^2^ is 22.74%.  Figure 4 presents the ROC curve for this model.

**Figure 5:**
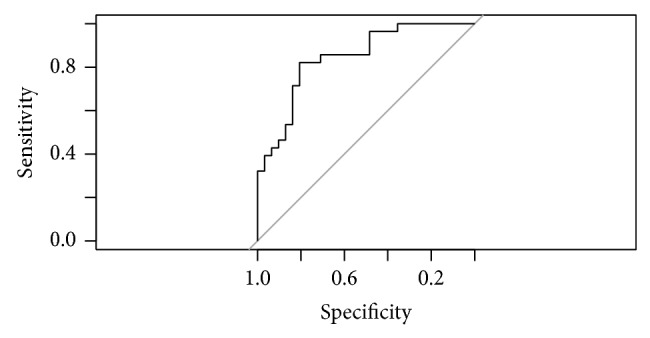
ROC curve for model MOD-B2.

MOD-B2 results present the effect of including the corneal variables along with MRA outcome: an increase of the diagnostic capacity of MOD-B1: OR = 2.79; 95% CI: 1.11–7.88. Apart from MRA, other predictors showed discriminating capacity between POAG and normality (Table 2). AUC of this model was 0.845 (95% CI: 0.746–0.943). Sensitivity is 71.43%; specificity is 81.75%. Nagelkerke *R* ^2^ is 44,92%.  Figure 5 presents the ROC curve for this model.

**Figure 6:**
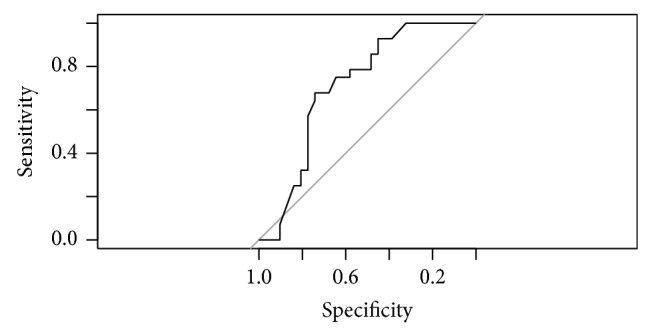
ROC curve for model MOD-C1.

MOD-C1 proved that GPS outcome has also significant diagnosis capability for glaucoma: OR = 1.36; 95% CI: 1.05–4.8. No significant interaction between age or disc size and the result of MRA analysis arose. AUC of this model was 0.712 (95% CI: 0.575–0.849). Sensitivity is 67.86%; specificity is 74.19%. Nagelkerke *R* ^2^ is 20,02%.  Figure 6 presents the ROC curve for this model.

**Figure 7:**
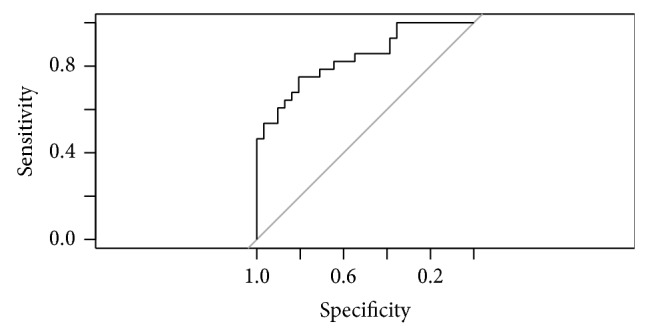
ROC curve for model MOD-C2.

MOD-C2 results presents, for another time, that the effect of including the corneal variables increases the diagnostic capacity of GPS alone: OR = 1.63; 95% CI: 1.09–3.28. Apart from GPS, other predictors showed discriminating capacity between POAG and normality (Table 2). AUC of this model was 0.838 (95% CI: 0.736–0.939). Sensitivity is 78.57%; specificity is 80.65%. Nagelkerke *R* ^2^ is 44,07%.  Figure 7 presents the ROC curve for this model.

Table 2 resumes the parameters presented above.

**Figure 8:**
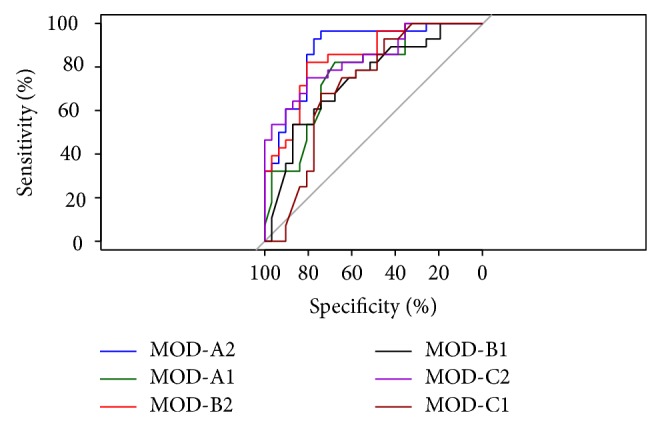
Comparison of ROC curves of all models.

Figure 8 presents a comparison of the ROC curves of all the models.

According to a DeLong's test for two correlated ROC curves, a statistically significant difference between AUC of MOD-A1 and MOD-A2 indeed exists (*Z* = 2.138; *P* = 0.032); the same occurs between MOD-B1 and MOD-B2 (*Z* = 1.965; *P* = 0.049) and between MOD-C1 and MOD-C2 (*Z* = 2.01; *P* = 0.044).

According to a logistic univariate regression analysis, the diagnostic accuracy of MOD-A1 depends on the stage of glaucoma (Hodapp, Parrish, and Anderson's classification: normality, early stage, and moderate glaucoma) (OR = 4.21; 95% CI: 1.7–12.12). The same phenomenon affects MOD-A2 (OR = 3.79; 95% CI: 1.55–10.69), MOD-B1 (OR = 2.44; 95% CI: 1.07–6.08), MOD-B2 (OR = 3.7; 95% CI: 1.53–10.29), and MOD-C2 (OR = 3.02; 95% CI: 1.28–8.04). However, the stage of glaucoma did not show a statistically significant influence on MOD-C1 (OR = 1.87; 95% CI: 0.84–4.51).

## 4. Discussion

Stereoscopic assessment of the optic nerve head by an expert glaucomatologist remains the gold standard for structural glaucoma diagnosis [1–11]. Nevertheless, and despite the former, the accuracy achieved by experts when evaluating ONH is by far higher than those of general ophthalmologist [1–11] and, what is more, proper glaucoma diagnosis must rely on the ONH evaluation along with a careful assessment of the visual field, IOP and CCT measurements, and a comprehensive ophthalmic examination [1–11]. Moreover, the access to stereoscopic photography of the ONH is not always viable in routine clinical practice [1–11].

During recent years we have witnessed an impressive technological development in the field of glaucoma structural analysis, which has resulted in many new diagnostic devices, such as the confocal laser ophthalmoscopy (Heidelberg Retina Tomograph) and optical coherence tomography in its various forms. Despite their evident utility, sophisticated analysis software, and flamboyant printouts given by these instruments, none of them provides pathognomonic results [12, 13] though their utility as ancillary diagnostic tools has, nowadays, become unquestionable [12–19].

Apart from being an excellent source of imaging of the ONH (and also the peripapillary RNFL) [20, 21], as a matter of fact, confocal laser scanning ophthalmoscope (Heidelberg Retina Tomograph) has proved to be an outstanding instrument for the diagnosis and follow-up of glaucoma [20]. Sanchez-Galeana et al. [21] reported an overall sensitivity and specificity ranging from 64% to 75% and from 68% to 80%, respectively. Ferreras et al. [22] claimed that the MRA global classification had a sensitivity of 73.9% and a specificity of 91.5%, while the GPS global classification had a sensitivity of 58.2% and a specificity of 94.4%. Ferreras et al. [22] study also put forward that the GPS had slightly higher sensitivity and somewhat lower specificity than the MRA when there was mild damage indicated by visual field tests and that the MRA had the best discrimination capability for moderate and severe glaucoma [22]. They also found that both the GPS and MRA had lower sensitivity and higher specificity for small optic discs (<1.7 mm^2^) compared with medium and large discs [22]. Nonetheless, the former results are far away from being homogeneous. Thus, Healey et al. [23] reported that the MRA sensitivity was 64.1%, specificity 85.7%, positive predictive value 21%, and negative predictive value 97.6% for detecting POAG. They also highlighted the interesting fact that including borderline results improved sensitivity (87.0%) but specificity dropped to 70.6% [23]. Moreover, as disc size increased, specificity fell, whereas sensitivity, POAG prevalence, and the proportion testing positive rose [23]. Andersson et al. [24] reported that the results of research comparing MRA and GPS are to some extent conflicting, particularly regarding sensitivity. Of all published studies, about 30% showed significantly or only slightly better sensitivity with MRA, around 50% indicated better sensitivity with GPS, and the remaining 20% demonstrated similar sensitivity for both methods [24]. Considering specificity, they claimed that a majority of the investigators showed that MRA was superior to GPS [24]. To sum up, a heterogeneous range of results regarding the diagnostic accuracy of the HRT could be found amongst different publications in the literature; however, more or less, the ranges are within the bounds we have quoted before [25–28].

Another issue which concerns the HRT and specifically the MRA is the fact that this test, along with the computing of the stereometric parameters of the ONH, relies on the contour line that must be established subjectively by the examiner himself. A great amount of attention has been paid to this [29–33] and despite the fact that a variation in the positioning of the contour line could have a certain impact on the determination of stereometric and diagnostic parameters, it does not seem to reach great signification. Moreover, the newish version of the HRT (HRT-III) which incorporates the GPS, providing a result independent of the contour line traced by the examiner, which relies on the analysis of the morphometry of the ONH and the peripapillary RNFL, has failed to significantly overtake MRA accuracy [24].

On the other hand, it is a well-known fact that glaucomatous population tends to have thinner corneas. What is more, CCT arises not only as a confounder for IOP measurements, but also as an independent risk factor for glaucoma [25–48]. It is somehow astonishing that while great attendance has been given to the analysis of the ONH and RNFL, the studies regarding the corneal structure characteristics inherent to glaucomatous eyes are quite few. In this vein, in several previous studies, we performed a corneal thickness segmentation scheme into virtual circular zones, concentric with the corneal apex, and we analyzed their differences between a sample of healthy volunteers and glaucomatous patients. As many differences arose between both samples, we also studied the diagnostic ability of our virtual segmentation model finding that, without being a diagnostic tool in itself, it showed a nondespicable capacity to discriminate between glaucomatous and healthy eyes [49–53].

Our findings left us with some questions whose answers went far beyond the purposes and capacities of those studies as they go far beyond this one: is there any structural difference between the cornea of glaucomatous eyes and the cornea of healthy eyes? In this case, is this difference primary or does it occur as a consequence of the disease itself? As an answer to the second question, we can say that CCT tends to be a steady parameter along the life of a person [25–53] but, in fact, we still do not have any information about the behaviour of the different corneal zones generated in our segmentation scheme throughout any period of time.

Taking the former into account, the very aim of this clinical study is to determine if adding the variables of the corneal thickness segmentation model to those of the HRT could improve its diagnostic accuracy. Our findings suggest that combining the analysis of both corneal and ONH structures indeed improves POAG diagnostic capability. To what extent this improvement could have any clinical impact is yet to be established.

## Figures and Tables

**Table 1:** Mean and standard deviation (std. deviation) of the corneal variables.

Report				
	Glaucoma cases versus healthy controls			
	Controls		Cases	
	Mean	Std. deviation	Mean	Std. deviation
CCT (ultrasound)	564.16	30.47	545.75	34.38
Overall corneal thickness	660.85	30.64	662.75	60.45
Zone I thickness	566.12	31.69	572.62	46.55
Zone II thickness	575.14	30.23	583.18	47.62
Zone III thickness	598.20	28.32	607.97	51.56
Zone IV thickness	635.11	29.15	643.24	58.57
Zone V thickness	683.62	35.91	687.02	64.43
Zone VI thickness	746.67	44.15	749.15	71.63

**Table 2:** Parameters showing the diagnostic ability of each significant variable in each model, respectively, (OR: odds ratio) and the diagnostic capacity of each model (sensitivity, specificity, positive and negative predictive values, AUC of ROC curves, and Nagelkerke *R* ^2^).

Model	Significant predictors	OR	OR 95% CI		AUC	Sensitivity (%)	Specificity (%)	Positive predictive value (%)	Negative predictive value (%)	Nagelkerke *R* ^2^ (%)
			Lower bound	Higher bound						
A1	ONH assessment	4.83	1.23	15.44	0.711	67.86	74.19	70.37	71.87	29.01

A2	ONH assessment	9.43	5.11	12.81	0.88	78.57	80.65	78.71	84.14	52.14
	Zone I	0.94	0.098	0.97						
	Zone III	1.33	1.19	1.49						
	Zone V	1.1	1.01	1.13						
	Zone VI	1.2	1.19	1.28						
	OT	0.85	0.69	0.87						

B1	ONH assessment	2.15	1.08	6.04	0.736	64.29	73.28	69.23	69.69	22.74

B2	ONH assessment	2.79	1.11	7.88	0.845	71.43	81.75	76.92	75.76	44.92
	Zone I	0.97	0.94	0.99						
	OT	0.98	0.96	0.99						

C1	ONH assessment	1.36	1.05	4.8	0.712	67.86	74.19	65.52	70.01	20.02

C2	ONH assessment	1.63	1.09	3.28	0.838	78.57	80.65	75.11	75.41	44.07
	Zone I	0.97	0.95	0.98						
	OT	0.89	0.76	0.95						
